# Hyaluronic acid–targeted topotecan liposomes improve therapeutic efficacy against lung cancer in animals

**DOI:** 10.3389/fonc.2024.1520274

**Published:** 2024-12-16

**Authors:** Gangqiang Xue, Lu Tang, Xinyan Pan, Sanni Li, Juan Zhao

**Affiliations:** ^1^ Department of Pharmaceutic Preparation, The Fourth Hospital of Shijiazhuang, Shijiazhuang, Hebei, China; ^2^ Institute of Food Science and Technology, Chinese Academy of Agricultural Sciences, Beijing, China; ^3^ Department of Endocrinology, Hebei General Hospital, Shijiazhuang, Hebei, China; ^4^ Department of Pharmacy, Hebei Children’s Hospital, Shijiazhuang, Hebei, China; ^5^ Experimental Center for Teaching, Hebei Medical University, Shijiazhuang, Hebei, China

**Keywords:** hyaluronidase, topotecan, liposomes, lung cancer, pharmacokinetics

## Abstract

Lung cancer, as a serious threat to human health and life, necessitating urgent treatment and intervention. In this study, we prepared hyaluronic acid (HA)-targeted topotecan liposomes for site-specific delivery to tumor cells. The encapsulation efficiency, stability, chemical structure, and morphology of HA-targeted topotecan liposomes were studied, and the release properties, cellular uptake capacity, and therapeutic efficacy of topotecan were further investigated. Results found that the coupling efficiency of HA on the surface of PEG-coated liposomes was determined to be 13.65 nmol/mg of lipid. The HA-targeted topotecan liposomes demonstrated a high encapsulation efficiency of 95% for topotecan, with an average particle size of 98.26 nm and excellent storage and dispersion stability. Drug release and cellular experiments indicated that the coating of HA further reduced the release rate of topotecan and decreased the survival rate of A549 cells, respectively. Flow cytometry and fluorescence staining analyses revealed that the HA-targeted topotecan liposomes enhanced the uptake of topotecan and exhibited significant anti-tumor effects on A549 cancer cells transplanted in mice. H&E staining showed that the pathological tissue treated with HA-targeted topotecan liposomes corresponded to Miller-Payne grade IV. Furthermore, these liposomes increased the accumulation of topotecan in tumors and extended the blood circulation time of the drug. Therefore, HA-targeted topotecan liposomes can be used as a new and easily prepared carrier in the field of lung chemotherapy, demonstrating considerable potential for anti-tumor therapy.

## Graphical Abstract

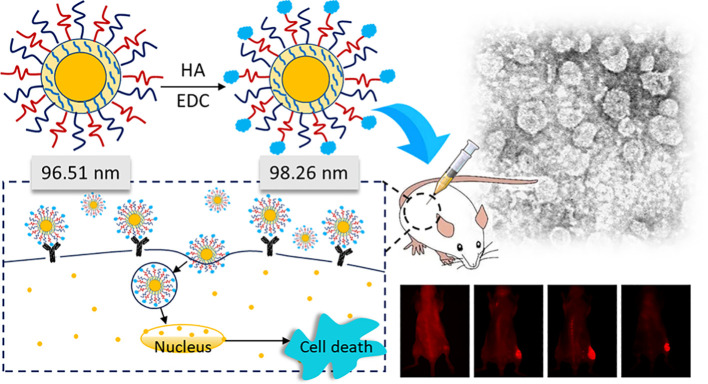

## 1 Introduction

Nowadays, lung cancer, as a malignant tumor originating in the lungs, is the most frequently diagnosed cancer worldwide (1, 2). Over the past several decades, the number of patients diagnosed with lung cancer has sharply increased globally. consequently, the treatment of lung cancer has become a research hotspot (3). Currently, low-dose computed tomography, immunotherapy, and targeted therapies are the primary methods for treating lung cancer (1, 4). However, their further application has been limited by side effects, instability, and unclear drug targets (5). Recently, targeted nanocarrier systems with low toxicity, good bioavailability, and high stability emerged as a promising strategy. These systems can enhance the selectivity of drugs towards tumors through specific guiding mechanisms and efficiently deliver drugs to target organs, thereby overcoming the limitations of passive targeting and reducing the side effects of medications on the body (6).

As spherical vesicles composed of one or more phospholipid bilayers, liposomes exhibit excellent intra-hydrophilic and inter-hydrophobic properties, which facilitate beneficial for the *in vivo* transport of encapsulated drugs and enhance their stability of drugs. Compared to other delivery systems, the structure of liposomes closely resembles that of cell membranes, thereby minimizing the toxicity associated with traditional encapsulation agents and demonstrating promising biodegradability, biocompatibility, and non-immunogenicity (7, 8). Liposomes have received clinical approval for cancer treatment due to their high payload capacity (9). Fuurthermore, liposomes can display targeted properties by modifying the surface molecules, enabling targeted drug delivery and the regulation of pharmacokinetics and biological distribution (10). Research has shown that nanoliposomes can evade uptake by the reticuloendothelial system due to the reduced recognition by circulating regulators (11). Additionally, the modification of polyethylene glycol (PEG) lipids can also inhibit liposome-induced complement activation (12, 13). Traditional PEG-modified long-circulating nanoliposomes can extend the circulation time of anti-tumor drugs in the bloodstream and enhance their accumulation in cancer cells (14, 15). To further improve the anti-tumor efficacy of liposomes, researchers have proposed some novel modification methods for PEG-coated liposomes, among which surface grafting of hyaluronic acid (HA) is a promising strategy (4, 9, 16). As an amphiphilic polysaccharide, HA can target and bind to overexpressed CD44 receptors on tumor cells (17, 18). The highly specific binding, facilitated by hydrogen bonding and electrostatic interactions between the amino acid residues of CD44 and the hydroxyl and carboxyl groups on the surface of HA, constructs a unique three-dimensional structure that promotes cell survival, growth, invasion, and metastasis through signaling networks (19).

Meanwhile, as a weakly basic camptothecin, topotecan is classified as a topoisomerase inhibitor. It can be loaded into liposomes using gradient-based loading methods and subsequently released slowly from these liposome carriers, inducing apoptosis in cell by mediating DNA strand breaks (20, 21). Clinically, it is utilized in the treatment of ovarian cancer, small-cell lung cancer, and cervical cancer (11, 12). This promising drug exhibits a short half-life, high bioavailability, and a low plasma protein binding rate. It has been approved by the FDA for second-line treatment of small-cell lung cancer, demonstrating significant potential for targeting tumors with drug-loaded liposomes (9, 22). However, current concerns include further stabilizing the encapsulation of topotecan, improving selectivity towards tumor cells, and enhancing drug uptake.

Therefore, in this study, HA was modified on the surface of PEG liposome to enhance drug delivery and improve the efficacy of tumor treatment. The topotecan encapsulated in the HA-targeted liposomes effectively reached the surface of tumor cells and was internalized to induce cell death. Furthermore, the preparation of HA-targeted topotecan liposomes and their mechanism of action are discussed. Subsequently, the efficacy of lung cancer treatment and the pharmacokinetics of HA-targeted topotecan liposomes are further evaluated.

## 2 Materials and methods

### 2.1 Materials

HA, egg phosphatidylcholine (EPC), and cholesterol were purchased from Sigma-Aldrich Corporation Co., Ltd. (Beijing, China). Polyethylene glycol-distearoylphosphatidylethanolamine (PEG2000-DSPE) and 1,2-distearoyl-sn-glycero-3-phosphoethanolamine-N-[carboxy-(polyethylene glycol)2000] (COOH-PEG2000-DSPE) were purchased from Avanti Polar Lipids, Inc. (USA). Ammonium sulfate and 1-ethyl-3-(3-dimethylaminopropyl) carbodiimide (EDC) topotecan hydrochloride were purchased from Jiangsu Henrui Medicine Co., Ltd. (Jiangsu, China). Hematoxylin and eosin staining solution was purchased from Coolaber Science & Technology Co., Ltd. (Beijing, China). The Hyaluronan Enzyme-Linked Immunosorbent Assay Kit was purchased from Echelon Industries Corporation (USA).

### 2.2 Preparation of topotecan liposomes

EPC, cholesterol, PEG2000-DSPE, and COOH-PEG2000-DSPE at different ratios (55:35:4:1, 55:40:4:1, 55:45:4:1, 55:50:4:1) were dissolved in chloroform, which was then evaporated to dryness under vacuum. The resulting lipid film was hydrated with 250 mM ammonium sulfate by sonication in a water bath for 5 min, followed by ultrasonication for 10 min. The suspensions obtained after hydration were successively extruded through polycarbonate membranes (Millipore, Bedford, MA, USA) with pore sizes of 400 nm and 200 nm for 3 times each to obtain blank PEG-coated liposomes. These liposomes were subsequently dialyzed several times in physiological saline. The loading of topotecan was achieved using the ammonium sulfate gradient-loading method. Specifically, topotecan hydrochloride was mixed with the blank PEG-coated liposomes for 30 min at 50°C in a water bath, and the obtained samples were referred to as PEG-coated topotecan liposomes (4).

**Figure 1:**
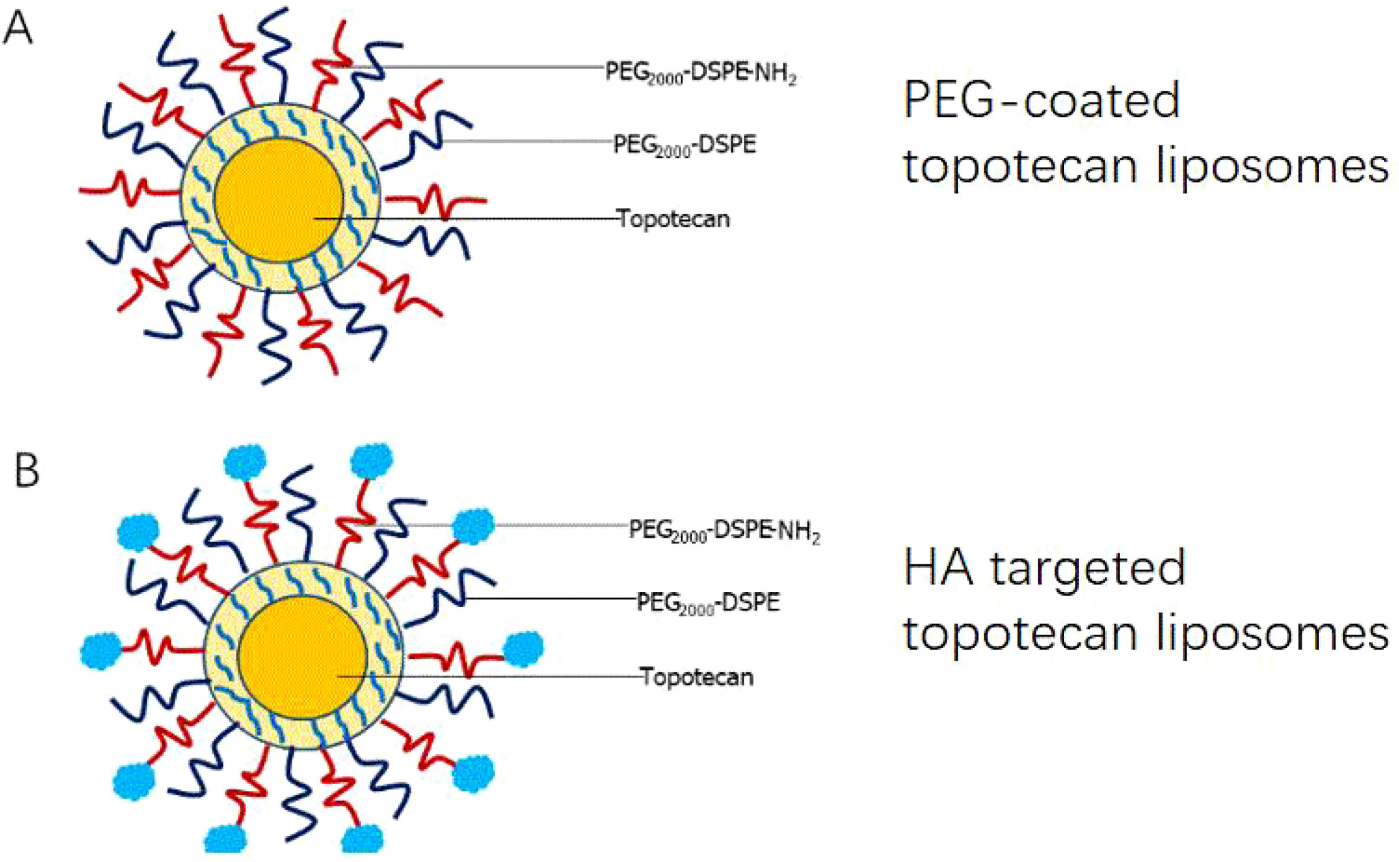
Schematic representations of two liposomal constructs consisting of PEG-coated topotecan liposomes **(A)** and HA-targeted topotecan liposomes **(B)**.

According to the description by Tiwari et al. (23), EDC was utilized as a coupling agent to prepare HA-targeted topotecan liposomes. Briefly, 4 mg of HA was dissolved in 1 ml of PBS (pH 7.4) and EDC was added to activate the carboxylic groups of HA. Subsequently, PEG-coated topotecan liposomes were incorporated at a lipid-to-ligand ratio of 1:0.04 (w/w) and incubated for 2 h incubation at room temperature to obtain HA-targeted topotecan liposomes. Additionally, the unreacted HA was removed through three rounds of centrifugation followed by washing with PBS (pH 7.4). The structural schematic diagrams of PEG-coated topotecan liposomes and HA-targeted topotecan liposomes are shown in **Figures 1A** and **B**, respectively.

### 2.3 Characterization of HA-targeted topotecan liposomes

The polymer dispersity index (PDI), zeta potential, and size distribution of the samples were measured using a zeta potential analyzer (Zetasizer Nano ZS, Malvern, England). Typically, the samples were dispersed in water (5 mL) for detection. PEG-coated topotecan liposomes and HA-targeted topotecan liposomes were passed through a Sephadex G-50 column (Sigma Aldrich Corporation, Beijing local agent, China) to eliminate any free topotecan. The topotecan present in the collected liposomes was quantified using a fluorescence spectrophotometer (Agilent Technologies Inc., Cotati, CA, USA). The fluorescence detection was set at λ_ex_ 370 nm and λ_em_ 530 nm. The above detection indicators were measured on the 1st, 3rd, and 7th days at 4°C, respectively. The encapsulation efficiency (EE) of topotecan was calculated as follows (11):


(1) $$\mathrm{EE}\;\left(\%\right)=\left({\text{W}}_{1}/{\text{W}}_{0}\right)\times 100\%$$

where W_1_ is the measured amount of topotecan in the liposomal suspensions after passing over the column, and W_0_ is the initial added amount of topotecan.

A Hyaluronan Enzyme-Linked Immunosorbent Assay Kit was used to measure the coupling efficiency of HA. First, 100 μL of standards or samples and 50 μL of the working detector were added to the incubation plate, which was then incubated for 1 h at 37°C. Following incubation, the mixtures (100 μL) were transferred to a detection plate and incubated for 30 min at 4°C. Next, 300 μL of 1×wash concentrate was used to wash the solution from the detection plate for four times to ensure complete removal of the wash buffer. Then, 100 μL of the working enzyme was added to the detection plate and incubated for 30 min at 37°C. The wash step was repeated and then 100 μL of the working substrate solution was added for 30-min reaction. The absorbance of the samples was measured at 405 nm, while the blank and standard curve were also assessed. The chemical structure of liposomes was detected using an fourier transform infrared spectrometer (FTIR) (Tensor 27, Bruker, Germany). Microscopic images of the prepared HA-targeted topotecan liposomes were obtained using a cryo-transmission electron microscopy (TEM) (Glacios, Thermo Fisher, USA).

### 2.4 Culture of A549 cells and cytotoxicity detection

According to the description by Wang et al. (24), DMEM (Sigma-Aldrich Corporation, Beijing local agent, China) was supplemented with 10% heat-inactivated fetal bovine serum (Sigma-Aldrich Corporation, Beijing local agent, China), 100 units/mL of penicillin (Sigma-Aldrich Corporation, Beijing local agent, China), and 100 units/mL of streptomycin (Sigma-Aldrich Corporation, Beijing local agent, China) was used for culture medium preparation. The cells were incubated at 37°C in an atmosphere containing 5% CO_2_. For cytotoxicity detection, A549 cells were seeded at a density of 9.0×10^3^ cells per well into sterile 96-well culture plates and cultured in culture medium in the incubator at 37°C with 5% CO_2_ for 24 h. Free topotecan (0-5 μM), PEG-coated topotecan liposomes (0-5 μM), and HA-targeted topotecan liposomes (0-5 μM) were then added to the 96-well culture plates. The survival rate was assessed after 48 h using the sulforhodamine B (SRB) staining assay, and the absorbance was recorded on a microplate reader (Spectra MAX190, Molecular Devices, USA) at 540 nm, according to the following formula (25):


(2) $$\mathrm{Survival}\;\mathrm{rate}\;\left(\%\right)=\;\left({\text{A}}_{1}/{\text{A}}_{2}\right)\times 100\%$$

where A_1_ is the absorbance value of the treated cells at 540 nm, and A_2_ is the absorbance value of the control cells at 540 nm.

### 2.5 Flow cytometry and *in vitro* binding specificity studies

According to Zhang et al. (9), approximately 6×10^5^ A549 cells per well were seeded into 6-well plates 24 h prior to measurement. The cells were incubated at 37°C for 1 h with free topotecan (1.5 μM), PEG-coated topotecan liposomes (1.5 μM), and HA-targeted topotecan liposomes (1.5 μM). The collected cells were trypsinized, pelleted by centrifugation, and washed three times with a PBS solution at 4°C. They were then analyzed using a flow cytometer (FACScan, Becton Dickinson, San Jose, CA), which excited at 488 nm and detected emissions at 560 nm. Additionally, the collected cells were fixed with 4% paraformaldehyde at room temperature for 20 min. After three washes with PBS, the cytoskeleton was stained with green fluorescently labeled phalloidin (immunohistochemistry), and the nucleus was stained with 4’, 6-diamino-2-phenylenediamine (DAPI). The fluorescence signal of topotecan was detected using an Axio Observer (Celldiscoverer 7, Zeiss, Germany) (4).

### 2.6 *In vivo* inhibition of orthotopic tumor growth

Female BALB/c nude mice and female Sprague-Dawley (SD) rats were purchased from the Beijing Vital River Laboratory Animal Technology Company. The animal study protocol was approved by the medical ethics committee of Shijiazhuang Obstetrics and Gynecology Hospital/The Fourth Hospital of Shijiazhuang (approval number: 20210067). Female BALB/c nude mice (18-20 g) were utilized to investigate the anti-tumor efficacy *in vivo*. Approximately 2.0×10^7^ A549 cell suspensions (2.0×10^7^ cells/ml) were prepared in Matrigel Matrix (BD Biosciences) for the establishment of orthotopic xenografts. The mice were anesthetized and positioned in the right lateral decubitus position. A 1 ml syringe fitted with a 29-gauge needle was used to inject 2×10^6^ cells percutaneously into the right lateral thorax at the lateral dorsal axillary line. The mice were randomly divided into treatment and control groups (six mice in each group) for intravenous administration of different samples, including physiological saline (control), free topotecan, PEG-coated topotecan liposomes, and HA-targeted topotecan liposomes, each at concentration of 3.5 mg/kg via the tail vein, administered after 16 days of A549 cell implantation. Tumor growth volume changes were non-invasively monitored using magnetic resonance imaging (MRI) over the course of one month. After 31 days, the tumors were weighed, and their tissues were sliced for H&E staining. The tumor volume was calculated as follows (26):


(3) $$\mathrm{Tumor}\;\mathrm{volume}=\mathrm{Length}\;\times \;\mathrm{Widt}{\text{h}}^{2}/2$$

During the process, when the diameter of subcutaneous tumors on thighs was measured to be higher than 5 mm, the *in vivo* near-infrared (NIR) imaging at 1, 4, 12, and 24 h was obtained using a maestro imaging system (CRi, Inc, Woburn, MA) by administering PGN-L-800CW via the tail vein.

### 2.7 Pharmacokinetics

Female Sprague-Dawley (SD) rats were used to analyze pharmacokinetics. Free topotecan, PEG-coated topotecan liposomes, and HA-targeted topotecan liposomes, each at a concentration of 3.5 mg/kg, were administered intravenously given via the tail vein. Plasma samples (1 mL) were collected in heparinized microcentrifuge tubes from the retroorbital venous sinus at 15 min, 30 min, 1 h, 2 h, 4 h, 8 h, 12 h, 24 h, and 48 h post-administration. The plasma was separated by centrifugation at 12,000 rpm for 10 min and 20 μL of the supernatant was injected into a sample bottle. A high-performance liquid chromatograph (HPLC) with fluorescence detection (Waters Technologies Inc., Cotati, CA, USA) was employed for measurement. Topotecan was separated using a Diamonsil C18 column (200×4.6 mm, 5 µm). The mobile phase consisted of acetonitrile and water containing 2.1 (w/v) citric acid and 0.2 (w/v) triethylamine (40/60, v/v) with a flow rate of 1.0 mL/min under isocratic conditions, and pH was adjusted to 3.8 using 30% sodium hydroxide. The fluorescence detection was set at λ_ex_370 nm and λ_em_ 530 nm (27).

### 2.8 Statistics

All experimental data were recorded following three parallel experiments. The results were analyzed by SPSS 22.0 statistics, after which *post hoc* tests with the Bonferroni correction were employed for multiple comparisons between individual groups. A P-value of ≤0.05 was considered significant by using Duncan’s multiple range tests.

## 3 Results and discussion

### 3.1 Characterization of HA targeted topotecan liposomes

The preliminary characterizations of traditional PEG-coated topotecan liposomes and HA-targeted topotecan liposomes are documented in **Table 1**. Generally, the encapsulation of topotecan by HA-targeted liposomes is influenced by the concentration gradient of ammonium sulfate inside and outside the liposomal membrane. This gradient facilitates the entry of topotecan into the liposomal vesicles, where it becomes ionized or precipitated, making it challenging for the drug to cross the membrane again and return to the external aqueous phase. As a result, this mechanism enhances the encapsulation efficiency of topotecan (28). The study found that the encapsulation efficiencies of topotecan in both types of liposomes exceeded 95% at a lipid ratio of 55:45 (EPC: cholesterol), indicating an optimal balance between the flexibility and stability of the liposomes, as regulated by cholesterol (29). After seven days of storage, the encapsulation efficiency of both liposome formulations showed no significant changes, demonstrating that the prepared liposomes possess good encapsulation stability. Furthermore, the modification of ligands (HA) did not significantly affect the encapsulation of topotecan, with a coupling efficiency of HA measured at 13.65 ± 2.11 nmol/mg lipid.

**Table 1:** Effect of the ratio of EPC to PEG2000-DSPE cholesterol to on the encapsulation efficiency of topotecan.

The ratio of EPC to cholesterol (μmol/μmol)	Encapsulation efficiency of topotecan	
	PEG-coated topotecan liposomes (%)	HA targeted topotecan liposomes (%)
55:35	88.35 ± 2.29	90.26 ± 2.93
55:40	91.39 ± 2.55	92.65 ± 2.42
55:45	95.26 ± 3.19	96.81 ± 2.66
55:50	93.16 ± 3.20	93.80 ± 3.05

As shown in **Table 2**, the mean sizes of fresh PEG-coated topotecan liposomes and HA-targeted topotecan liposomes were 96.51 ± 3.08 nm and 98.26 ± 2.01 nm, respectively. The zeta potentials were -28.59 ± 0.68 mV and -25.29 ± 0.50 mV, indicating that the prepared samples showed good dispersion stability (30, 31). A slight increase in liposome size was found due to the coating with HA, which was also observed in the results reported by Ravar et al. (6). Notably, the stability of the topotecan-loaded liposomes showed no significant changes during the 7-day storage period, demonstrating excellent storage stability and promising potential for future applications. These results are consistent with previous studies on folate-targeted topotecan liposomes (4).

**Table 2:** Characterizations of two topotecan liposomes.

Day	Detection indictor	PEG-coated topotecan liposomes	HA targeted topotecan liposomes
1	Encapsulation efficiency of topotecan (%)	95.26 ± 3.19	96.81 ± 2.66
	Mean size (nm)	96.51 ± 3.08	98.26± 2.01
	Zeta-potential	-28.59 ± 0.68	-25.29 ± 0.50
	PDI	0.15 ± 0.01	0.13 ± 0.01
3	Encapsulation efficiency of topotecan (%)	95.35 ± 3.12	96.85 ± 3.09
	Mean size (nm)	96.77 ± 2.20	98.12 ± 2.99
	Zeta-potential	-28.31 ± 1.15	-25.16 ± 0.92
	PDI	0.15 ± 0.01	0.14 ± 0.01
7	Encapsulation efficiency of topotecan (%)	95.38 ± 2.88	96.89 ± 2.56
	Mean size (nm)	96.89 ± 3.02	98.30 ± 2.63
	Zeta-potential	-28.26 ± 1.52	-25.12 ± 1.03
	PDI	0.16 ± 0.01	0.15 ± 0.01

**Figure 2:**
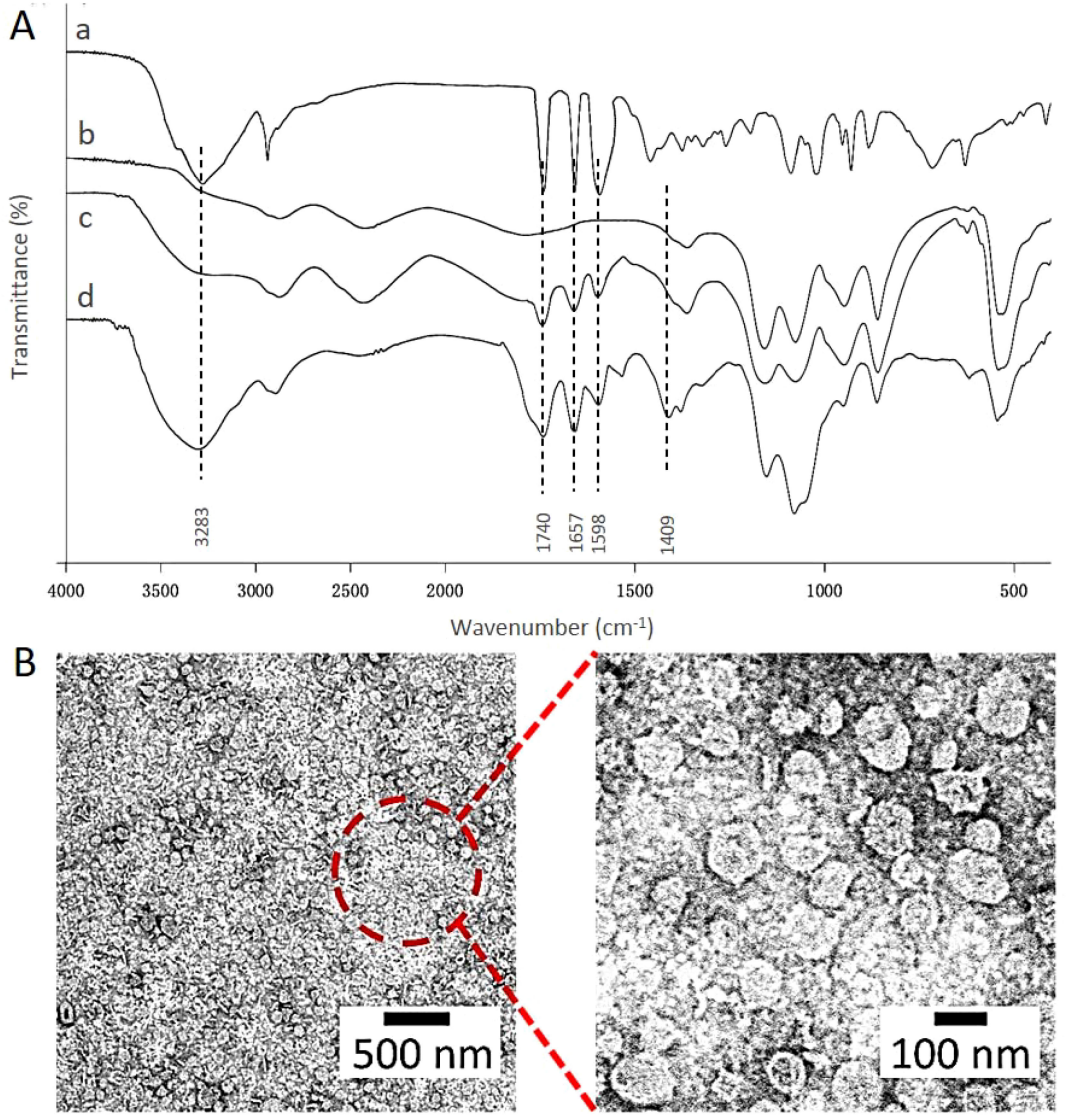
**(A)** FTIR spectra of HA **(a)**, the control liposomes **(b)**, PEG-coated topotecan liposomes **(c)**, and HA-targeted topotecan liposomes **(d)**. **(B)** Morphology of HA-targeted topotecan liposomes.

FTIR spectra further confirmed the encapsulation of topotecan in HA-targeted liposomes (**Figure 2A**). In the FTIR spectrum of topotecan, the strong peak at 3283 cm^-^¹ corresponds to O-H stretching (32). Typical peaks at 1740 cm^-^¹, 1657 cm^-^¹, and 1598 cm^-^¹ were assigned to the stretching vibrations of C=O, C-N, and C=C, respectively (33). After loading topotecan, the spectra of PEG-coated topotecan liposomes and HA-targeted topotecan liposomes exhibited distinct absorption features from topotecan, demonstrating the effective loading of the drug in the liposomes. Furthermore, the characteristic absorption of C=O in HA was also observed in HA-targeted topotecan liposomes, indicating the successful grafting of HA (34).

Cryo-TEM was employed to examine the micro-morphology of HA-targeted topotecan liposomes, as illustrated in **Figure 2B**. All HA-targeted topotecan liposomes were uniformly distributed and exhibited spherical shapes, with no abnormal liposomes observed. This indicates that the coupling of HA and the drug embedding did not alter the structural characteristics of liposomes. Furthermore, the size of the HA-targeted liposomes was measured to be approximately 100 nm. Additional examination of this figure revealed no significant aggregation in the distribution of liposomes, which aligns with the high zeta potential mentioned earlier.

### 3.2 Release and delivery properties

**Figure 3:**
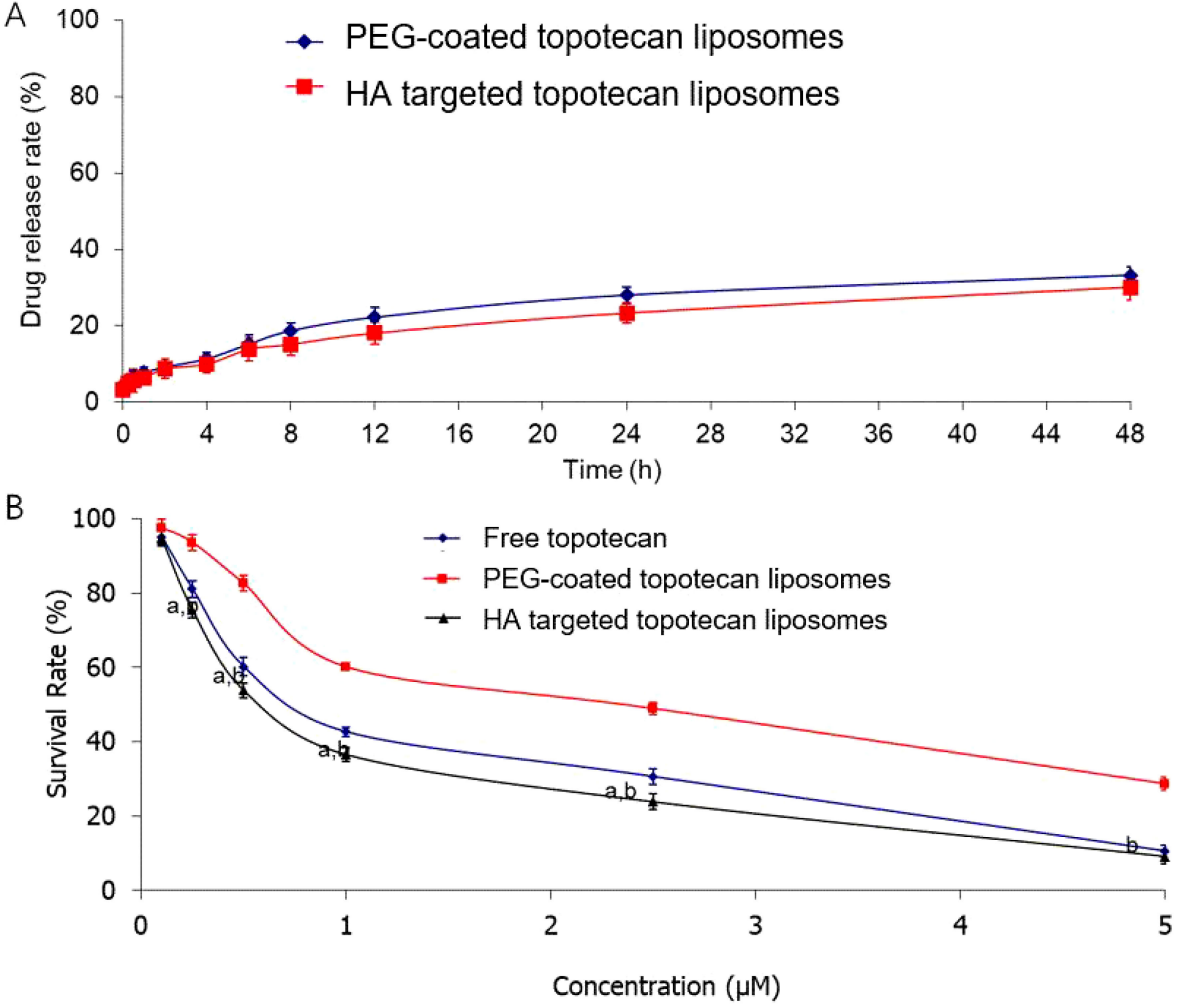
**(A)** The release profiles of two different liposomes in PBS solution containing 10% serum protein at 37 °C. Data are presented as the mean ± SD (n=3). **(B)** Survival rates of A549 cells after applying various topotecan formulations measured by SRB staining assay. Data are presented as the mean ± S.D. (n=3). a, P<0.05, versus free topotecan; b, P<0.05, versus PEG-coated topotecan liposomes.

As shown in **Figure 3A**, the drug release rates of the two types of liposomes were analyzed. Compared to PEG-coated topotecan liposomes, the HA-targeted topotecan liposomes showed a lower release rate. Following the grafting of HA, the PEG-HA coating formed a denser aqueous layer on the surface of the liposomes, which more effectively stabilized the lipid bilayer structure and created steric hindrance, thereby further inhibiting the release of topotecan (35).

The relatively low *in vitro* release rate (<30%) could be overlooked due to the excellent therapeutic efficacy of the samples. The cytotoxicity of HA-targeted topotecan liposomes containing topotecan was investigated *in vitro* using A549 cells. **Figure 3B** shows that HA-targeted topotecan liposomes are more effective at killing A549 cells compared to others. This enhanced efficacy is attributed to the targeted binding between HA and CD44 in tumor cells, which increases the drug delivery efficiency, as previously mentioned. The ranking of the survival rates for A549 cells at different concentrations was HA-targeted topotecan liposomes< PEG-coated topotecan liposomes< free topotecan.

### 3.3 Flow cytometry studies

**Figure 4:**
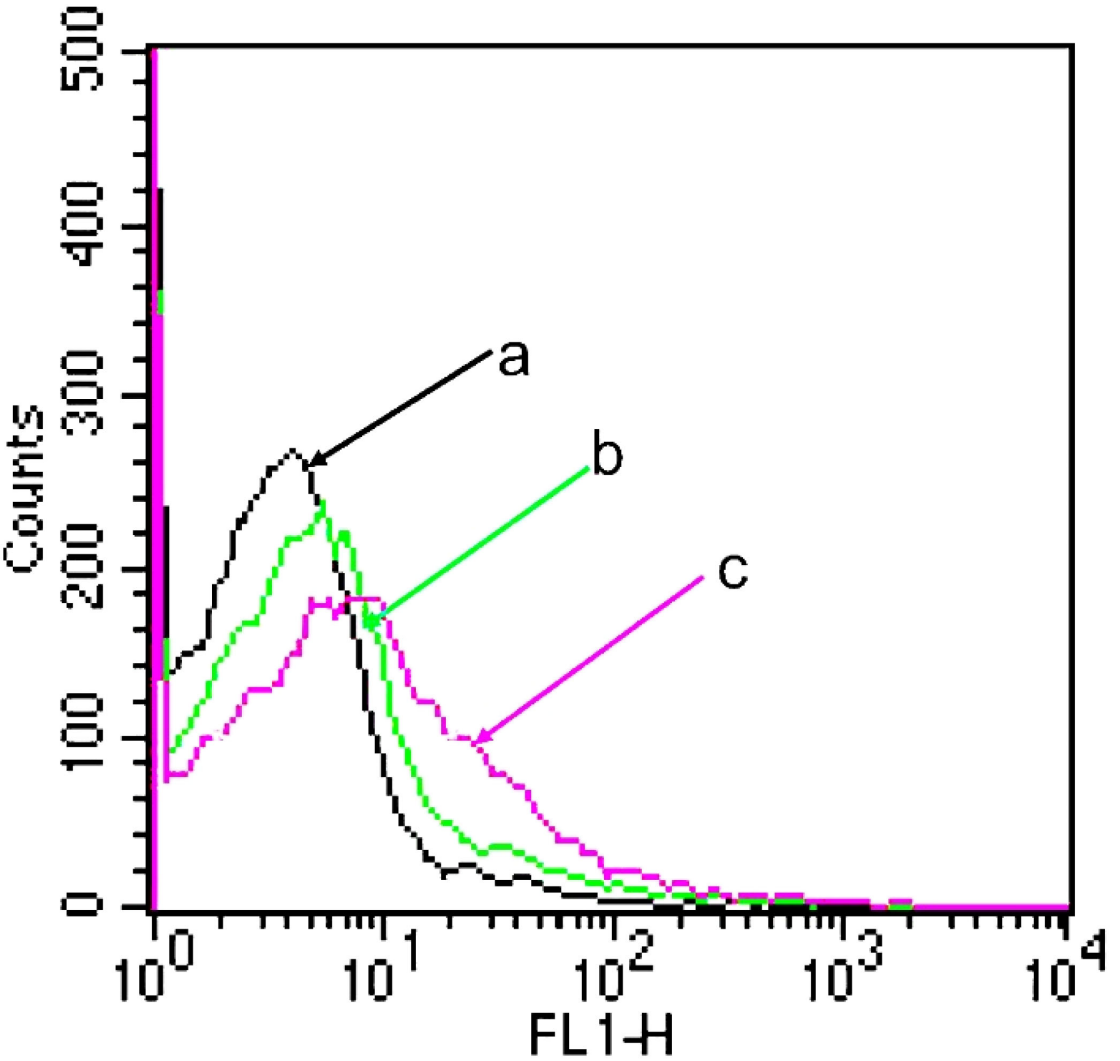
A549 cellular uptake after applying various topotecan formulations measured by flow cytometry. **(A)** PBS; **(B)** PEG-coated topotecan liposomes; **(C)** HA-targeted topotecan liposomes.

**Figure 4** shows the kinetic uptake of topotecan by A549 cancer cells at 37°C. In general, a higher uptake of drugs by cells correlates with increased fluorescence intensity (36). The data presented in this figure indicate that HA-targeted topotecan liposomes showed the highest fluorescence intensity among all samples. This suggests that the endocytosis of HA-targeted topotecan liposomes by the cells was more pronounced, thereby enhancing their efficacy in killing A549 cancer cells. This effect is closely associated with the targeting capability of HA.

**Figure 5:**
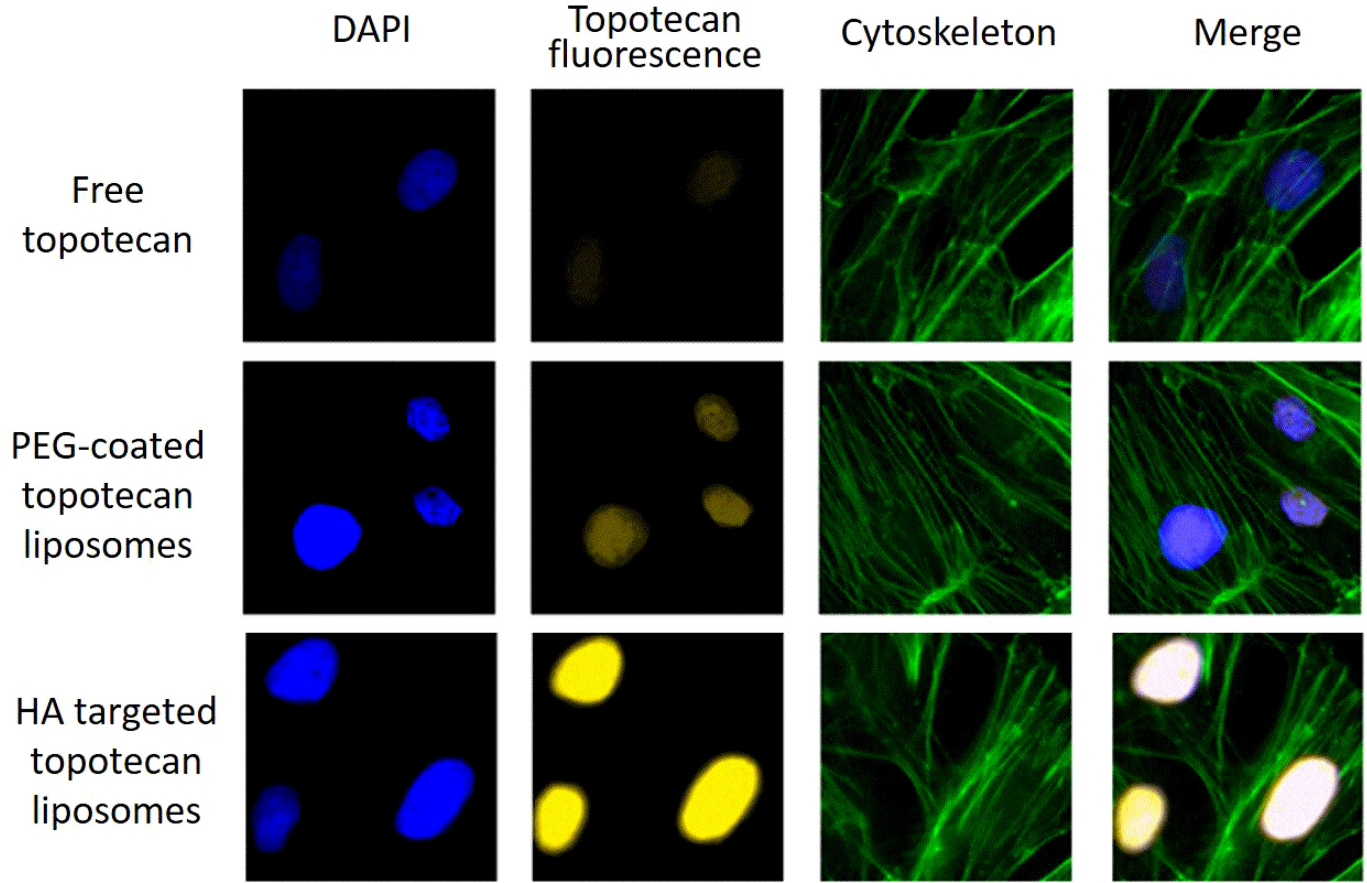
Drug distribution in A549 cells following the application of free topotecan, PEG-coated topotecan liposomes, and HA-targeted topotecan liposomes. blue represents DAPI-stained cell nucleus, yellow represents topotecan-stained cell nucleus, green represents cytoskeletal actin-stained cells.

Furthermore, **Figure 5** displays the binding specificity of topotecan to support the above analysis. The nucleus, labeled with blue fluorescence via DAPI staining, represents the successful uptake of liposomes into the cells (37). In comparison to free topotecan treatment, both PEG-coated topotecan liposomes and HA-targeted topotecan liposomes treatment showed higher signal intensity, illustrating the uptake of a greater number of liposomes. Besides, the yellow fluorescence intensity in A549 cells treated with HA-targeted topotecan liposomes was further enhanced, representing a more targeted enrichment of topotecan into the interior of cells. The increased cellular uptake of HA-targeted topotecan liposomes may be attributed to the interaction between HA and CD44, which activates the cytoplasmic domain of CD44. This interaction induces the recruitment of neutrophils and initiates a series of cellular signaling events, leading to the rearrangement of the cytoskeleton for tumor invasion and migration (19). Notably, the green signal corresponding to the cytoskeletal structure among the three groups was clear and intact, with no obvious destruction observed. These findings indicated that A549 cells treated with HA-targeted topotecan liposomes showed the highest intake efficiency and targeted drug delivery capability without toxicity.

### 3.4 *In vivo* inhibition of orthotopic tumor growth

**Figure 6:**
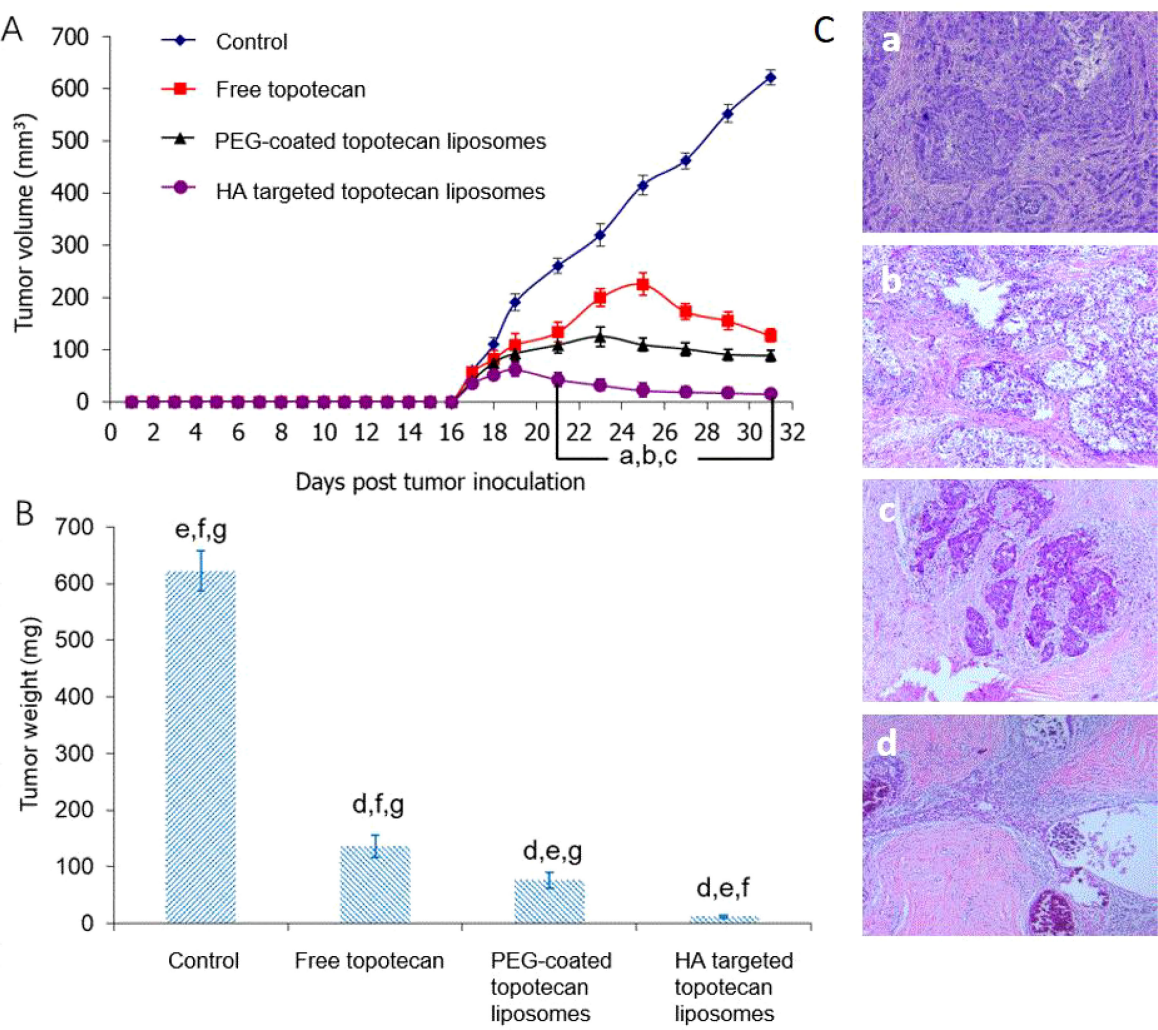
**(A)** Effect of HA-targeted topotecan liposomes on the A549 cell xenografts in female nude mice. At 17, 20, 23, 26, and 29 days after inoculation, physiological saline as control group, free topotecan, PEG-coated topotecan liposomes, and HA-targeted topotecan liposomes were given intravenously to mice via tail vein. Data are presented as the mean ± SD (n=6). a, P<0.05, versus physiological saline; b, P<0.05, versus free topotecan; c, P<0.05, versus PEG-coated topotecan liposomes. b, Tumor weight on the 31st day. d, P<0.05, versus physiological saline; e, P<0.05, versus free topotecan; f, P<0.05, versus PEG-coated topotecan liposomes; g, P<0.05, versus HA-targeted topotecan liposomes. c, H&E staining images of tumor tissue treated by physiological saline **(a)**, free topotecan **(b)**, PEG-coated topotecan liposomes **(c)**, and HA-targeted topotecan liposomes **(D)**.

As shown in **Figure 6**, the anti-cancer effects of HA-targeted topotecan liposomes were further investigated on orthotopic tumor-bearing nude mice following the injection of A549 cells. The tumor volume (**Figure 6A**) and tumor weight (**Figure 6B**) were recorded. During the first 16 days, tumor volume was monitored across various treatment groups and the blank control, revealing no obvious increase, which indicates the absence of notable toxic or side effects. On the 17th day, it was observed that the tumors had reached a sufficient mass for treatment. By the 31st day, significant differences in tumor volume were noted among the different samples, with HA-targeted topotecan liposomes exhibiting a greater inhibitory effect on tumor growth. The ranking of the inhibitory effects was HA-targeted topotecan liposomes> PEG-coated topotecan liposomes> free topotecan> blank control, which corresponded to the cytotoxicity of the different samples against A549 cells. Notably, from the 19th day onward, the targeted delivery of HA-targeted topotecan liposomes had a therapeutic effect on tumors, resulting in a reduction in tumor volume. This effect was attributed to the enhanced delivery efficiency of topotecan, which played an important role in the inactivation of tumor cells (17). The evaluation of tumor weight further supported this discussion. As shown in **Figure 6B**, on the 31st day, tumors treated with intravenously injected HA-targeted topotecan liposomes exhibited the lightest weight compared to the other samples, consistent with the analysis of tumor volume changes.

Afterward, the number of residual cancer cells identified in pathology was used to determine the efficacy of HA-targeted topotecan liposomes (**Figure 6C**). Generally speaking, the pathological specimens were evaluated using the Miller/Payne (MP) grading system, which primarily assesses the proportion of tumor cell reduction, ranging from I to V. Higher MP grades indicate better therapeutic efficacy (38). It was found that the pathological tissue treated with HA-targeted topotecan liposomes showed a limited presence of invasive cancer cells, corresponding to MP grade IV. In contrast, the reduction of cancer cells in other treatment groups was less than 90% (MP grade≤III), suggesting that HA-targeted topotecan liposomes have the potential to enhance therapeutic efficacy against lung cancer.

### 3.5 *In vivo* longitudinal NIR optical imaging of PGN-L-800CW

**Figure 7:**
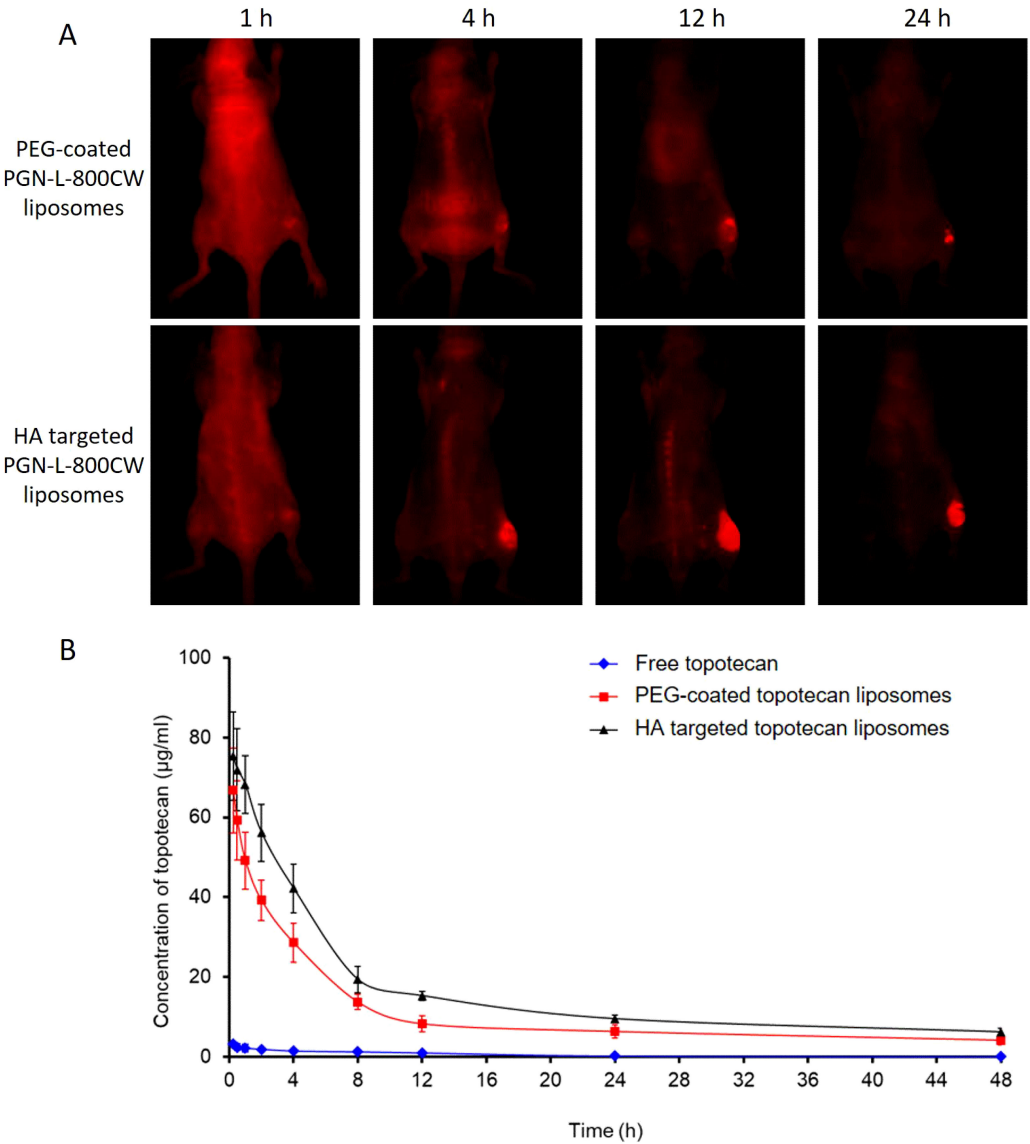
**(A)** *In vivo* NIR imaging of PGN-L-800CW after intravenous administration of PEG-coated Aur-L-800CW liposomes, and HA-targeted PGN-L-800CW liposomes for 1 h, 4 h, 12 h, and 24 h. **(B)** Time course of topotecan levels in plasma after intravenous administration of free topotecan, PEG-coated topotecan liposomes, and HA-targeted topotecan liposomes. Each point represents the mean ± SD (n=6). **(A)** P<0.05, versus PEG-coated topotecan liposomes; **(B)** P<0.05, versus HA-targeted topotecan liposomes.

PGN-L-800CW was used as a nanoprobe in NIR imaging to minimize interference from mouse fur at infrared wavelengths. PEG-coated PGN-L-800CW liposomes served as the control group to assess the effective uptake of HA-targeted PGN-L-800CW liposomes. As can be seen from **Figure 7A**, *in vivo* NIR imaging exhibited that no significant uptake of PGN-L-800CW was observed within the first hour following intravenous injection. However, after 4 h, the administration of HA-targeted PGN-L-800CW liposomes resulted in a distinct tumor contrast with minimal tissue background, which was significantly more pronounced than that of PEG-coated Aur-L-800CW liposomes. After 24 h, NIR imaging showed a higher PGN-L-800CW fluorescence in the tumor, likely due to the enhanced binding affinity of the HA-targeted delivery, which improved the targeting specificity of PGN-L-800CW and increased drug uptake efficiency, thereby enhancing signal intensity (37).

### 3.6 Pharmacokinetics

Pharmacokinetics primarily concerns the absorption, distribution, and elimination of drugs, and can be defined as the study of drug processes in both human and non-human animal bodies (39). Liposome drug delivery systems typically offer improved pharmacokinetics (7). The *in vivo* pharmacokinetics of various topotecan formulations were measured, and the results are presented in **Figure 7B**. Notably, the concentration of free topotecan in plasma rapidly decreased following intravenous injection in female SD rats, whereas HA-targeted topotecan liposomes and PEG-coated topotecan liposomes maintained higher concentrations during the initial stages and exhibited a more gradual decline. In comparison to PEG-coated topotecan liposomes, HA-targeted topotecan liposomes demonstrated a higher plasma topotecan level during the terminal phase, resulting in prolonged circulation in the bloodstream, attributed to the formation of protein crowns around the injected nanoparticles (40).

Moreover, a two-compartment model was employed to determine the pharmacokinetic parameters of topotecan following the intravenous administration of free topotecan, PEG-coated topotecan liposomes, and HA-targeted topotecan liposomes in female rats. As indicated in **Table 3**, HA-targeted topotecan liposomes exhibited higher half-life (t1/2 β), maximum concentration (Cmax), mean residence time (MRT), and area under the curve (AUC) compared to the other formulations. The initial half-life (t1/2 α) of free topotecan was significantly lower than that of PEG-coated and HA-targeted topotecan liposomes, while the clearance (CL) of free topotecan was significantly higher than that of others. This further supported the conclusion that the intravenous injection of HA-targeted topotecan liposomes enhanced drug concentration in plasma and improves the therapeutic efficacy in targeting lung tumors.

**Table 3:** Pharmacokinetic parameters of topotecan after intravenous administration of free topotecan, PEG-coated topotecan liposomes, and HA-targeted topotecan liposomes in female rats at a dose of 3.50 mg/kg.

Pharmacokinetic parameters	Free topotecan	PEG-coated topotecan liposomes	HA targeted topotecan liposomes
t1/2 α (h)	1.69± 0.36	2.95± 0.60	3.12 ± 0.69
t1/2 β (h)	7.55 ± 1.90	15.12 ± 3.16	19.05 ± 2.19
Cmax (μg/ml)	1.51 ± 0.22	53.89± 8.55	59.13± 8.07
CL (ml/h/kg)	2.99 ± 0.24	0.20± 0.03	0.22 ± 0.01
MRT_0-48h_ (h)	6.85 ± 1.12	9.96 ± 2.15	10.52 ± 1.89
AUC_0-48h_ (μg·h/ml)	10.9` ± 1.65	132.91± 15.83	146.03 ± 20.19

Data are presented as the mean ± SD (n = 6).

t1/2α (h), distribution half-life; t1/2β (h), elimination half-life; Cmax, peak concentration; CL (ml/h/kg), total body clearance; MRT0-48 h, mean residence time; AUC0-48 h (μg/ml·h), area under the plasma concentration–time curve.

## 4 Conclusions

In this study, a spherical HA-targeted topotecan liposome was successfully developed, demonstrating superior anti-lung tumor efficacy compared to traditional PEG-coated liposomes. These liposomes showed a high encapsulation efficiency for topotecan and a slower release rate of the drug. Additionally, topotecan could be absorbed more effectively and induce cancer cell death through receptor-mediated endocytosis, thereby reducing the risk of lung tumor progression. The liposome formulation was classified as MP-IV grade. *In vivo* NIR imaging further confirmed that HA-targeted topotecan liposomes delivered topotecan more precisely to the interior of tumor cells. Consequently, this liposome formulation represents a promising strategy for lung cancer therapy via receptor-mediated targeting, providing new insights for the treatment of lung cancer.

## Data Availability

The original contributions presented in the study are included in the article/supplementary material. Further inquiries can be directed to the corresponding author.
